# Direct Observation of Propagating Spin Waves in the 2D van der Waals Ferromagnet Fe_5_GeTe_2_

**DOI:** 10.1021/acs.nanolett.3c02212

**Published:** 2023-11-13

**Authors:** Frank Schulz, Kai Litzius, Lukas Powalla, Max T. Birch, Rodolfo A. Gallardo, Sayooj Satheesh, Markus Weigand, Tanja Scholz, Bettina V. Lotsch, Gisela Schütz, Marko Burghard, Sebastian Wintz

**Affiliations:** ‡ Max Planck Institute for Intelligent Systems, Heisenbergstrasse 3, D-70569 Stuttgart, Germany; ¶ Universität Augsburg, D-86159 Augsburg, Germany; § Max Planck Institute for Solid State Research, Heisenbergstrasse 1, D-70569 Stuttgart, Germany; ∥ RIKEN Center for Emergent Matter Science, JP-351-0198 Wako, Japan; ⊥ Universidad Técnica Federico Santa María, Avenida España 1680, 2390123 Valparaiso, Chile; # Helmholtz-Zentrum Berlin für Materialien und Energie GmbH, Hahn-Meitner-Platz 1, D-14109 Berlin, Germany

**Keywords:** spin dynamics, 2D magnets, spin waves, 2D spintronics, time-resolved X-ray microscopy

## Abstract

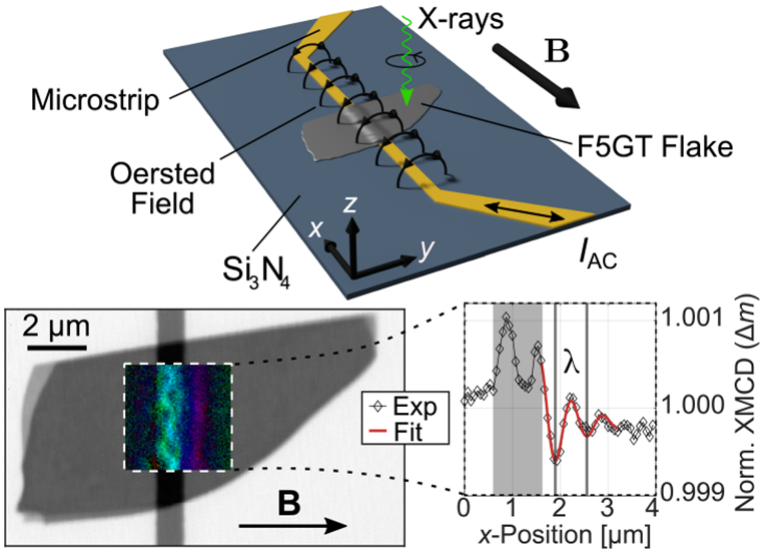

Magnetism in reduced dimensionalities is of great fundamental interest while also providing perspectives for applications of materials with novel functionalities. In particular, spin dynamics in two dimensions (2D) have become a focus of recent research. Here, we report the observation of coherent propagating spin-wave dynamics in a ∼30 nm thick flake of 2D van der Waals ferromagnet Fe_5_GeTe_2_ using X-ray microscopy. Both phase and amplitude information were obtained by direct imaging below *T*_C_ for frequencies from 2.77 to 3.84 GHz, and the corresponding spin-wave wavelengths were measured to be between 1.5 and 0.5 μm. Thus, parts of the magnonic dispersion relation were determined despite a relatively high magnetic damping of the material. Numerically solving an analytic multilayer model allowed us to corroborate the experimental dispersion relation and predict the influence of changes in the saturation magnetization or interlayer coupling, which could be exploited in future applications by temperature control or stacking of 2D-heterostructures.

The growing interest in magnetic 2D van der Waals (vdW) materials stems both from the possibility of studying fundamental physics in lower dimensions and from their potential applications in future technologies. Many physical phenomena manifest themselves differently in low dimensions, such as electron transport, optical-, as well as optoelectrical properties, which have been most carefully studied in graphene.^1,2^ With the recent discovery of magnetic 2D materials such as FePS_3_^3,4^ and Cr_2_Ge_2_Te_6_,^5^ it has been become feasible to study magnetic ordering in reduced dimensionality, down to the monolayer limit,^6^ providing insight into the underlying physics.

While many magnetic systems show interesting behavior in the 2D limit,^7^ most of them are not prime candidates for applications, as their Curie temperature *T*_C_ is far below room temperature. This is due to the fact that atoms close to a surface find a decreased number of nearest neighbors, reducing the effective stabilization by exchange and making the magnetically ordered state less stable against thermal fluctuations. Nevertheless, experimental works have revealed magnetically ordered states in 2D vdW magnets with a *T*_C_ above room temperature like bulk CrTe^8^ or even ultrathin Cr_2_Te_3_.^9^ It has been shown that the magnetic anisotropy plays an essential role in stabilizing magnetic order in these systems, especially because even a small magnetic anisotropy leads to the breakdown of the Mermin-Wagner-Theorem.^10,11^ One promising candidate for room temperature applications is naturally annealed Fe_5_GeTe_2_, which—depending on the growth method and Fe content—was found to exhibit a *T*_C_ between 270 and 330 K.^12−14^

The spin dynamics of 2D vdW systems is closely linked to the field of magnonics,^15−17^ where coherent spin waves are used as a low-dissipation information carrier, with both their phase and amplitude being utilizable for information processing. Spin dynamics in Fe_5_GeTe_2_ have been studied using ferromagnetic resonance (FMR) spectroscopy, focusing on the determination of the *g*-factor along different crystal axes and the temperature dependence of magnetic damping.^14^ In general, spin-wave propagation is expected to behave differently in a 2D vdW material compared to a typical bulk magnet,^18^ and has been studied in antiferromagnetic CrCl_3_ in the case of localized standing spin waves,^19,20^ and in CrI_3_ using spectroscopic techniques,^21^ or time-resolved magneto-optic Kerr microscopy.^22^ However, the direct observation of propagating spin waves in a magnetic 2D vdW material with both spatial and temporal resolution has not yet been reported.

In this work, we present spatially and temporally resolved magnetization measurements of propagating spin-wave dynamics in micrometer-sized flakes of Fe_5_GeTe_2_ with a thickness of ∼28 nm, recorded at low temperatures using time-resolved scanning transmission X-ray microscopy (TR-STXM). Here, the X-ray magnetic circular dichroism (XMCD) effect allowed us to measure the out-of-plane magnetization component *m*_*z*_, while we gained temporal resolution via an asynchronous pump–probe scheme.^23^ The spin-wave dynamics measured by this method provided access to simultaneous phase and amplitude information in both the backward-volume and Damon–Eshbach geometry. This further allowed us to determine parts of the spin-wave dispersion relation for frequencies between 2.77 and 3.84 GHz with corresponding wavelengths from 1.5 to 0.5 μm. The results were compared to a theoretical model based on a multilayer approach in order to determine various system parameters and gain insight into the saturation magnetization and interlayer coupling dependence of the spin-wave dispersion relation.

Fe_5_GeTe_2_ single crystals were synthesized in quartz glass ampules from the pure elements Fe, Ge, and Te in a 6:1:2 ratio in the presence of iodine acting as a vapor transport agent. The vacuum-sealed ampules were heated to a temperature of 750 °C at a rate of 120 °C/h and kept at that temperature for 2 weeks before letting them slowly cool inside the furnace. This *naturally annealed* type of Fe_5_GeTe_2_ has different properties with regards to anisotropy behavior compared to the more commonly used *quenched* Fe_5_GeTe_2_, where the crystal is rapidly cooled after growth.^13^ Here, we focus on slow-cooled Fe_5_GeTe_2_ as the quenched type was found to exhibit an irreversible change of magnetic properties at low temperature and significant variations in local anisotropy.^24^ Elemental composition was confirmed by energy dispersive X-ray spectroscopy using a Tescan SEM Vega TS 5130 MM instrument equipped with a silicon drift detector, yielding a value of Fe_4.93(6)_GeTe_2.00(2)_.

Fe_5_GeTe_2_ grown this way is known to possess rhombohedral symmetry, best described by the R3̅m space group (No. 166).^25^ It consists of two-dimensional sheets of Fe and Ge sandwiched between layers of Te, as shown in Figure 1a. The unit cell, with *a* = *b* = 4 Å and *c* = 29 Å, spans over three vdW layers, with a gap size of *h* = 3 Å. Within one layer, the atoms are held together by covalent bonds, but the individual layers interact mainly via vdW forces.

**Figure 1:**
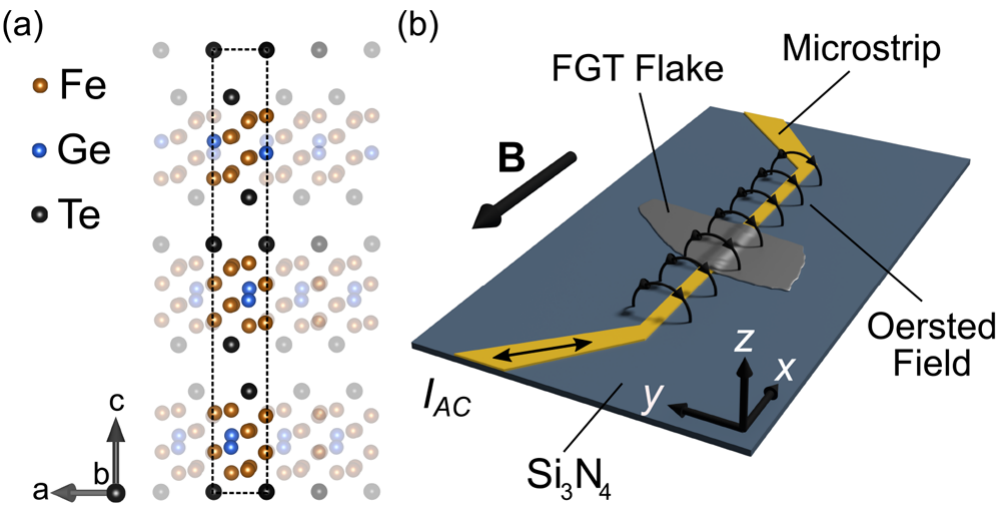
(a) Crystal structure of Fe_5_GeTe_2_, with the unit cell marked by the dashed line containing three vdW layers. (b) Sample sketch, Fe_5_GeTe_2_ flake on top of the patterned microstrip, all on a silicon nitride (Si_3_N_4_) membrane window on a SiO_*x*_ frame. External static field **B**, as well as the Oersted field generated by the alternating current (*I*_*AC*_) are indicated by black arrows.

The *T*_*c*_ of the bulk crystal was determined to be 285 K using SQUID magnetometry as shown in the Supporting Information. This value is indicative of crystallographic Fe_5_GeTe_2_ rather than Fe_3_ GeTe_2_ with local Fe inclusions, for which lower *T*_*c*_ values would be expected. There are three different Fe sites in the crystal lattice of Fe_5_GeTe_2_, out of which two are magnetically ordered at the temperatures considered in this work.^13^

For the dynamical measurements, a 1 μm wide Au strip of 50 nm thickness was microstructured onto an X-ray-transparent Si_3_N_4_ membrane using e-beam lithography and thermal evaporation in a lift-off approach. The microstrip was then covered with 50 nm of MgO for electrical insulation using ion beam sputtering. The Fe_5_GeTe_2_ crystal is first cleaved, and then flakes of different thickness are exfoliated using scotch tape. A 28 nm thick flake of Fe_5_GeTe_2_ was then selected and exfoliated from the Scotch brand tape using a polydimethylsiloxane (PDMS) stamp, transferred onto the MgO covered microstrip, and subsequently covered by a hexagonal boron nitride (hBN) flake to prevent degradation under ambient conditions. Atomic force microscopy (AFM) measurements reveal a continuous bending of the flake over a distance of 0.5 to 1 μm on each side of the microstrip without any signs for sharp deformation apart from a slight kink of the flake close to the center of the microstrip. A sketch of the sample is shown in Figure 1b. This sample was then studied by TR-STXM at the MAXYMUS endstation at the BESSYII electron storage ring operated by the Helmholtz-Zentrum Berlin für Materialien und Energie. By exploiting the XMCD effect, time-resolved microscopy images of the dynamic out-of-plane magnetization component of the magnetically ordered Fe sites were obtained.

Figure 2a shows a static STXM image of the Fe_5_GeTe_2_ flake with the Au microstrip beneath visible as a horizontal bar. Near the edge of the flake, one can see stepwise thickness variations; however, these do not extend toward the center of the flake. To determine the Fe_5_GeTe_2_ thickness, the photon count *I* on the flake was compared to that of the surrounding area *I*_0_. The flake thickness was estimated by measuring the X-ray transmission at an off-resonant photon energy (1150 eV). Together with the energy-dependent X-ray absorption coefficient of Fe_5_GeTe_2_^26^ and Beer’s law, the thickness was estimated to be approximately 28 nm. This measurement was confirmed by additional AFM measurements. All dynamic measurements were performed at the Fe L_3_ edge and in a uniformly magnetized state (see Figure 2a), which was reached by applying a saturating in-plane magnetic field. The 4 × 4 μm^2^ area at the center of the flake, marked by dashed lines, indicates where the dynamic measurements were performed. At lower fields or different temperatures, also nonuniform spin textures such as stripe domains were observed by static imaging. The field strength required to reach the uniformly magnetized state showed a strong temperature dependence, with the magnetic states at higher temperatures being more readily saturated as a result of a temperature dependent magnetic anisotropy.^24,27^

**Figure 2:**
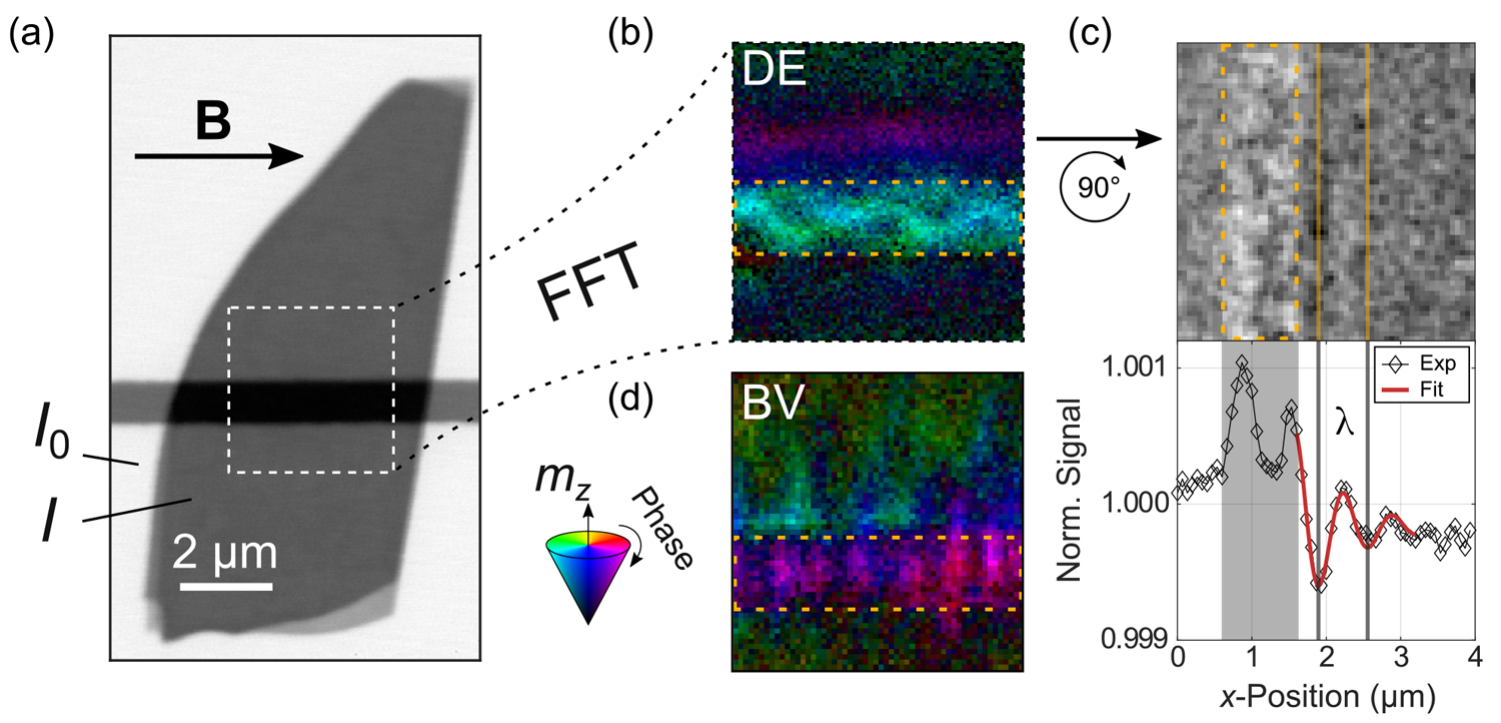
(a) Static STXM image of the Fe_5_GeTe_2_ flake with the dashed rectangle marking the position of the dynamic measurements. (b) Phase-resolved map of the spin-wave dynamics, recorded at a temperature of 190 K in an external field of 22.5 mT and an excitation frequency of 3.07 GHz. (c) Cross-section (top) of the dynamic measurement snapshot (bottom), recorded at 190 K, 22.5 mT, and 3.84 GHz with a decaying sine function (orange) fitted to the area adjacent to the microstrip. (d) Backward volume type spin waves recorded on a different flake of ∼50 nm thickness at nominal 160 K, 35 mT, and 3.84 GHz. The position of the microstrip is marked by dashed yellow lines in parts b–d.

Figure 2b shows the result of a TR-STXM measurement containing both phase and amplitude information. Multiple frames of the dynamically excited spin texture were recorded in a pump–probe setup with different time delays. This was done for each individual point of the scan area while maintaining the relative phase information, resulting in a movie of the magnetization dynamics. The frequency-filtered representation shown in Figure 2b is the result of performing a temporal fast Fourier transform (FFT) for each of the pixels, taking into account the information of all frames at once. The result is displayed in an HSV color scale, where the color (hue) indicates the relative phase of the signal, and its brightness (value) indicates the FFT amplitude, which is proportional to the out-of-plane magnetization component *m*_*z*_.^28^ The colored cone in the inset of Figure 2 illustrates how phase and amplitude are encoded. The data were recorded at a temperature of 190 K in an external field of 22.5 mT and for an excitation frequency of 3.07 GHz. More information on how the temperature was determined can be found in the Supporting Information, together with animated movies of all dynamic magnetization measurements.^27^

The dynamic image, as well as the animated movies, clearly show coherent Damon–Eshbach (DE) type spin waves (**k** ⊥ **M**) emitted from the Au microstrip and propagating through the Fe_5_GeTe_2_ flake. This emission shows a distinct asymmetry with respect to the microstrip, as can be seen in Figure 2b, an effect that has been observed before and is attributed to the relative orientation between the Oersted field around the microstrip and the local deflection of magnetization of the spin wave.^29^ This was confirmed in the experiment by changing the field direction by 180°, which resulted in a reversal of the main excitation direction.

Another noticeable feature is the inhomogeneity of the dynamic signal on top of the microstrip in the area marked by the dashed yellow box in Figure 2b. One possible origin for this inhomogeneity could be the presence of backward-volume (BV) type spin waves (**k**||**M**) that occur along the microstrip and might form such kinds of patterns, in which case the pattern should change as a function of frequency. However, such a frequency dependence was not observed in subsequent measurements at other frequencies, which are shown in Supporting Information. It is therefore more likely that local inhomogeneities, bending, the aforementioned kink, or strain lead to the formation of the pattern observed in Figure 2b. In Figure 2c, the cross-section of the out-of-plane magnetization component, taken along the direction of propagation for DE geometry, is shown together with a snapshot of the dynamic measurement. For better statistics, the signal was integrated along the axis of parallel wavefronts. The measurement was then repeated on the same flake, again at a temperature of 190 K and an external field of 22.5 mT, this time at an excitation frequency of 3.84 GHz. From this cross-section, the wavelength of the propagating spin wave and its decay length can be determined by fitting a function of the form *a* sin(*bx* + *c*)·exp(−*dx*) + *e* to the area adjacent to the microstrip, which yielded a wavelength of ∼0.6 μm, and a decay length of ∼0.7 μm for the example shown in Figure 2c. The signal-to-noise ratio did not always allow for this kind of approach, and hence, for some snapshots the wavelength was estimated manually without a fit function. From the decay length and a linearized group velocity of 800 m s^–1^, a spin-wave lifetime of τ = 875 ps ps is determined, leading to an estimated total line width of *τ f* = 0.3, in agreement with the FMR line width measured for bulk Fe_5_GeTe_2_ in ref (14) at 200 K. The relatively large line width (short decay length) indicates that the material has a high effective damping for spin waves compared to other materials commonly used in magnonic research, such as yttrium–iron-garnet and permalloy, where spin waves can travel up to several hundred microns.^30,31^

Figure 2d exemplifies BV type spin waves that have been observed in a different Fe_5_GeTe_2_ flake of ∼50 nm thickness at a nominal temperature of 160 K. A magnetic field of 35 mT was applied parallel to the microstrip, and the linear (not parametric) excitation frequency was 3.07 GHz. It can be seen that the BV type spin waves are mainly confined to the microstrip and only partially extend into the flake. The emergence of BV type spin waves in a DE field geometry has been observed before^32^ and can be attributed to local inhomogeneities of the flake or the microstrip, which may act as sources for spin-wave excitation.

Further TR-STXM measurements were performed under the same conditions as in Figure 2, parts b and c, except for using different excitation frequencies *f*, from which the spin-wave wavelengths were determined either manually or with a fit function. With these results, it was possible to experimentally determine parts of the spin-wave dispersion of the Fe_5_GeTe_2_ flake in the DE geometry, as shown in Figure 3a. Note that for all *k* values measured, the corresponding wavelength is already smaller than half of the width of the antenna, which means that the excitation occurs at higher harmonics of the antenna width. However, this has no significant effect on the analyzed spin-wave pattern and dispersion outside of the antenna region. In the investigated frequency range, the dispersion relation exhibits an almost perfect linear behavior, which one can see enlarged in the inset. The purple line in Figure 3a was calculated using a dynamic matrix approach based on the linearized Landau–Lifshitz equation; a method well suited for modeling the spin-wave dispersion and mode profiles of thick ferromagnetic layers, where the film thickness is several times the exchange length of the material. More information on the model can be found in the Supporting Information, as well as in the literature.^33^ It is worth noting that the multilayer approach is used to consider the variation of the dynamic magnetization along the film thickness, because such a thickness dependence is lost in a simple macrospin model. For the model parameters, initial values for Fe_5_GeTe_2_ were taken from literature.^12−14^ The experimental data were then fitted under a reasonable variation of parameters, yielding a saturation magnetization of *M*_s_ = 210 kA m^–1^, an exchange constant of *A*_ex_ = 9 pJ m^–1^, and an anisotropy field of *H*_a_ = 1.6 kA m^–1^. A list of all parameters used in the calculation can be found in Supporting Information. For the interlayer exchange coupling constant *J*, associated with the exchange interaction between spins along the normal direction, ab initio coupling parameters of Fe_3_GeTe_2_ were used as a starting point.^34^

**Figure 3:**
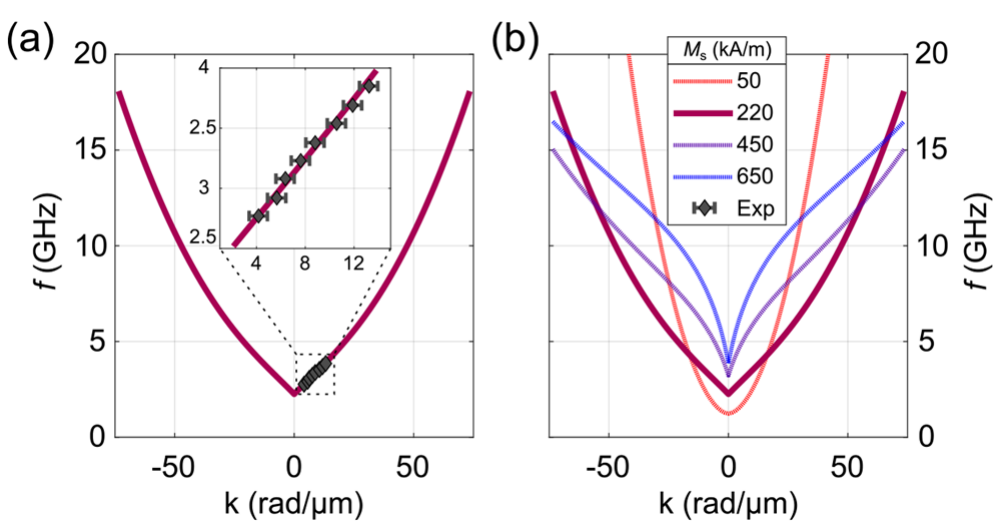
(a) Experimentally determined dispersion relation of Fe_5_GeTe_2_ (gray diamonds) with a theoretical fit (purple line) using a multilayer model. (b) Calculated dispersion relation of a 28 nm thick flake for different values of saturation magnetization *M*_s_.

The resulting overall shape of the dispersion relation, shown in Figure 3a, looks quite peculiar, since it does not have the typical DE type shape of common magnonics materials such as permalloy or yttrium–iron-garnet,^35,36^ which exhibit a steep increase in *f* for small *k*, followed by an intermediate region with a smaller slope, and ultimately a quadratic behavior for large *k* and thus small wavelengths. Typically, the small and large *k* regimes are dominated by dipolar and exchange interaction respectively, however, the curve obtained for Fe_5_GeTe_2_ does not show these distinct characteristics at the given scales.

To gain further insight into this particular spin-wave dispersion relation, calculations were made for different values of saturation magnetization *M*_s_, shown in Figure 3b. It is evident that for larger values of *M*_s_ (650 kA m^–1^), the dispersion relation shows the typical DE type behavior, but for small values of *M*_s_ (50 kA m^–1^), it is purely quadratic on the given scale. The dispersion therefore sensitively depends on the dipolar interaction, which in turn is proportional to *M*_s_^2^, i.e. for a system with low *M*_s_, dipolar interactions are negligible, and exchange interaction dominates even at large wavelengths. At 190 K, the Fe_5_GeTe_2_ flake has a relatively low *M*_s_ compared to many bulk materials used in magnonics and therefore exhibits a DE spin-wave dispersion relation between that of purely exchange- and dipolar interaction dominated systems.

With its Curie temperature *T*_C_ between 270 and 330 K,^12−14^ Fe_5_GeTe_2_ will show significant changes in *M*_s_ when the temperature is changed, leading to effects similar to those shown in Figure 3b. Although other properties will also change as a function of temperature, such as anisotropies and exchange stiffness, we argue that saturation magnetization will have the strongest impact on the spin-wave dispersion due to the quadratic increase of dipolar interaction with *M*_s_. While this fact would necessitate a precise control of temperature in devices based on this material, it also opens up the general possibility of tuning the dispersion relation in this material by means of temperature control. Another aspect to be considered regarding temperature variation is that of magnetic damping in Fe_5_GeTe_2_, which was found to be strongly temperature dependent in bulk crystal ferromagnetic resonance experiments.^14^

Apart from temperature dependencies, what makes magnetic 2D materials truly distinct from most 3D materials is their anisotropic exchange interaction. While most materials used in magnonics show a rather isotropic exchange interaction, 2D materials typically exhibit a strongly reduced interlayer exchange coupling constant *J* between the individual vdW layers compared to the lateral exchange, depending on both the stacking type and the size of the vdW gap. Using the multilayer model introduced earlier, it is possible to calculate the dispersion relation of Fe_5_GeTe_2_ for different interlayer coupling strengths. It turned out that the zeroth order spin-wave mode does not change significantly when *J* is varied (see Supporting Information), however, the higher-order modes are strongly affected by the value of *J*, as modeled in Figure 4a. There, the dispersion relation of the first higher-order spin-wave mode is shown for different interlayer coupling strengths. The mode profiles of the zeroth and first order DE type spin-wave modes are shown schematically in Figure 4c for the resonance case (uniform lateral precession *k* = 0). The first higher-order mode displays a precession node, with the node position for *k* ≠ 0 depending on *k*, as is also the amplitude decay over the film thickness for the zeroth mode.^35^ The mode profile provides a hint as to why the energy and therefore frequency of the higher-order mode depend on the interlayer coupling strength. Neighboring magnetic moments will have different precession angles, which for higher coupling strengths leads to a higher energy. In 2D materials, this causes a decrease in energy of higher-order spin-wave modes compared to isotropic systems, and Figure 4b shows how the *k* = 0 resonance frequency of the first higher-order mode *f*_k=0_^1^ changes as a function of *J*. A clear linear behavior is found with slight deviations at low values of *J*. Two points are marked on this line, namely the isotropic case, where *J* is chosen to match the lateral intralayer exchange coupling, and an exemplary value of a 2D system, which was calculated using the coupling constants of Fe_3_GeTe_2_ as obtained by DFT calculations^34^ in the absence of literature values for Fe_5_GeTe_2_. This underlines the principal possibility to determine the value of *J* by measuring the *k* = 0 resonance frequency of the higher-order mode and obtain information on the magnetic coupling between different layers in magnetic 2D vdW materials.^37,38^ It also demonstrates that by stacking different magnetic 2D materials, it may become possible to tailor higher-order spin-wave modes from the perspective of potential magnonic applications.

**Figure 4:**
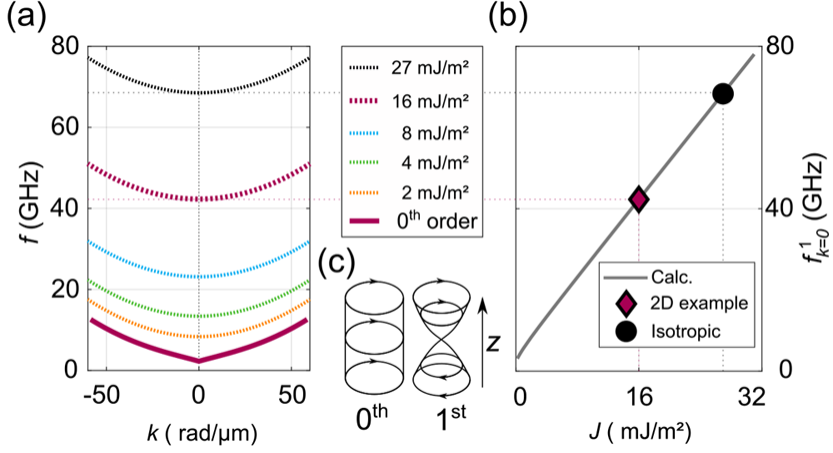
(a) First-order spin-wave modes for different interlayer coupling constants *J*. (b) *k* = 0 frequency of the first order spin-wave mode *f*_k = 0_^1^ as a function of *J*. The center (c) shows qualitative ferromagnetic resonance (*k* = 0) profiles of the 0th and 1st order modes. Calculated for a system with a thickness of 28 nm and, except for *J*, standard Fe_5_GeTe_2_ parameters.

To conclude, we directly imaged coherent propagating spin waves in a magnetic 2D material with both spatial and temporal resolution. Thin flakes of Fe_5_GeTe_2_ were studied with TR-STXM at a temperature of 190 K at different external fields and excitation frequencies. From the experimental data, it was possible to sample part of the dispersion relation at different frequencies. These data points were then used to model and fit the dispersion relation of Fe_5_GeTe_2_ by utilizing a multilayer approach based on the dynamic matrix method. This enabled us to study the system also for a range of saturation magnetization values, showing that the observed DE dispersion relation occurs in between the cases of exchange-dominated and magneto-dipolar governed dispersions. The model further allowed for calculating the dispersion relation of higher-order spin-wave modes as a function of interlayer coupling strength, revealing distinct characteristics of spin-wave dynamics of 2D materials as compared to 3D systems. Both the variation of *M*_s_ and *J* opens up the possibility of tuning the magnetic properties of 2D materials by means of temperature control and stacking, which could be exploited in future 2D spintronic applications.

## Data Availability

The data that support the findings of this study are available from the corresponding author upon request.

## Supporting Information Available

*M*(*T*) curve of Fe_5_GeTe_2_, a description of the temperature determination, snapshots and animated movies of the TR-STXM measurements, information on the computational model, and additional calculations in both DE and BV geometry (PDF)
Movie of the 2.77 GHz TR-STXM measurement (AVI)
Movie of the 2.92 GHz TR-STXM measurement (AVI)
Movie of the 3.08 GHz TR-STXM measurement (AVI)
Movie of the 3.23 GHz TR-STXM measurement (AVI)
Movie of the 3.38 GHz TR-STXM measurement (AVI)
Movie of the 3.54 GHz TR-STXM measurement (AVI)
Movie of the 3.69 GHz TR-STXM measurement (AVI)
Movie of the 3.84 GHz TR-STXM measurement (AVI)

## Supplementary Material

nl3c02212_si_002.pdf

nl3c02212_si_003.avi

nl3c02212_si_004.avi

nl3c02212_si_005.avi

nl3c02212_si_006.avi

nl3c02212_si_007.avi

nl3c02212_si_008.avi

nl3c02212_si_009.avi

nl3c02212_si_010.avi
