# Supplementary material for: Direct Observation of Propagating Spin Waves in the 2D van der Waals Ferromagnet Fe5GeTe2
Source: Nano Lett. 2023 Nov 13;23(22):10126–31. doi: 10.1021/acs.nanolett.3c02212 (PMC10683057; doi:10.1021/acs.nanolett.3c02212)
Supplement: Supplementary file 1 — nl3c02212_si_002.pdf [file nl3c02212_si_002.pdf]

Supporting Information: Direct Observation of Propagating Spin Waves in the 2D van-der-Waals Ferromagnet Fe_5GeTe_2

Frank Schulz,[*,†,†] Kai Litzius,[¶,†,‡] Lukas Powalla,[§] Max T. Birch,[‖,‡] Rodolfo A. Gallardo,[⊥] Sayooj Satheesh,[§] Markus Weigand,[‡,#] Tanja Scholz,[§] Bettina V. Lotsch,[§] Gisela Schütz,[‡] Marko Burghard,[§] and Sebastian Wintz[*,‡,#]

†*These authors contributed equally.*

‡*Max Planck Institute for Intelligent Systems, Heisenbergstrasse 3, D-70569 Stuttgart, Germany*

¶*Universität Augsburg, D-86159 Augsburg, Germany*

§*Max Planck Institute for Solid State Research, Heisenbergstrasse 1, D-70569 Stuttgart, Germany*

‖*RIKEN Center for Emergent Matter Science, JP-351-0198 Wako, Japan*

⊥*Universidad Técnica Federico Santa María, Avenida España 1680, 2390123 Valparaiso, Chile*

#*Helmholtz-Zentrum Berlin für Materialien und Energie GmbH, Hahn-Meitner-Platz 1, D-14109 Berlin, Germany*

E-mail: fschulz@is.mpg.de; sebastian.wintz@helmholtz-berlin.de

SQUID Magnetometry

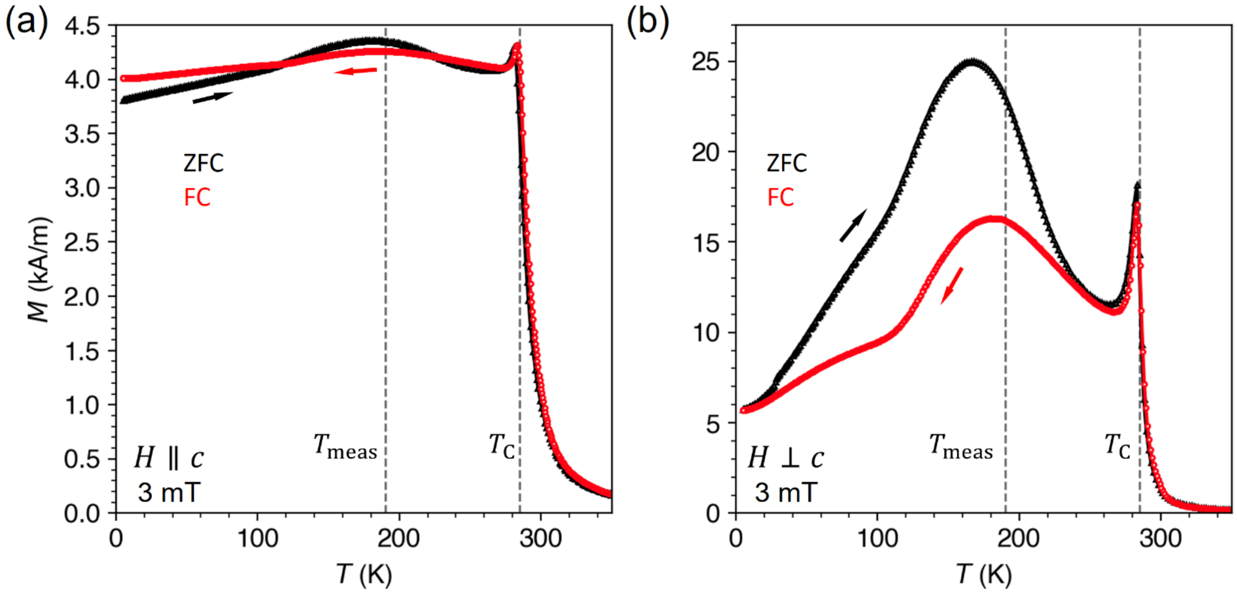

Supplementary Fig. S1: M(T) curves obtained using SQUID magnetometry on a bulk crystal of Fe_5GeTe_2 at an external field of 3 mT applied (a) parallel and (b) perpendicular to the c-axis of the crystal for a field cooled (FC, red) and zero field cooled (ZFC, black) procedure. The temperature at which the TR-STXM measurements were performed, as well as the Curie temperature T_C are marked by dotted and dashed lines, respectively. Note that M in general does not correspond to Ms, since the material is not magnetically saturated over a wide range of temperature at the given field.

Determining the Temperature T

For the determination of T during the TR-STXM measurements, the XMCD contrast was measured by detecting the photon flux I through the Fe_5GeTe_2 flake for both positive (C^+) and negative (C^-) circular x-ray polarizations, with the magnetization being saturated out-of-plane at 250 mT. Apart from the magnetic field, this measurement was taken using the standard experimental conditions, namely with the cryostat set to a specific temperature T_{set}^{RF}, and the alternating excitation current turned on. For the TR-STXM measurements of

the Damon-Eshbach type spin-waves, this e.g. yielded a ratio of

$$\frac{I(\mathrm{C}^{+}, T_{\mathrm{set}}^{\mathrm{RF}})}{I(\mathrm{C}^{-}, T_{\mathrm{set}}^{\mathrm{RF}})} = 0.933.$$

The RF current was then turned off, and the cryostate set to increasing temperatures $T_{\mathrm{set}}^{\mathrm{off}}$ until the previously determined XMCD contrast could be reproduced. For the 28 nm thin Fe_5GeTe_2 flake, this was the case at $T_{\mathrm{set}}^{\mathrm{off}} = 190\,\mathrm{K}$ (for a ratio of 0.933). Taking into account this heating of the sample by the RF current, we can identify the true temperature of the measurement, in this case $T = 190$ K.

TR-STXM Results

Animated movies of all TR-STXM measurements shown in Fig. S2 can be downloaded as *.avi* files separately.

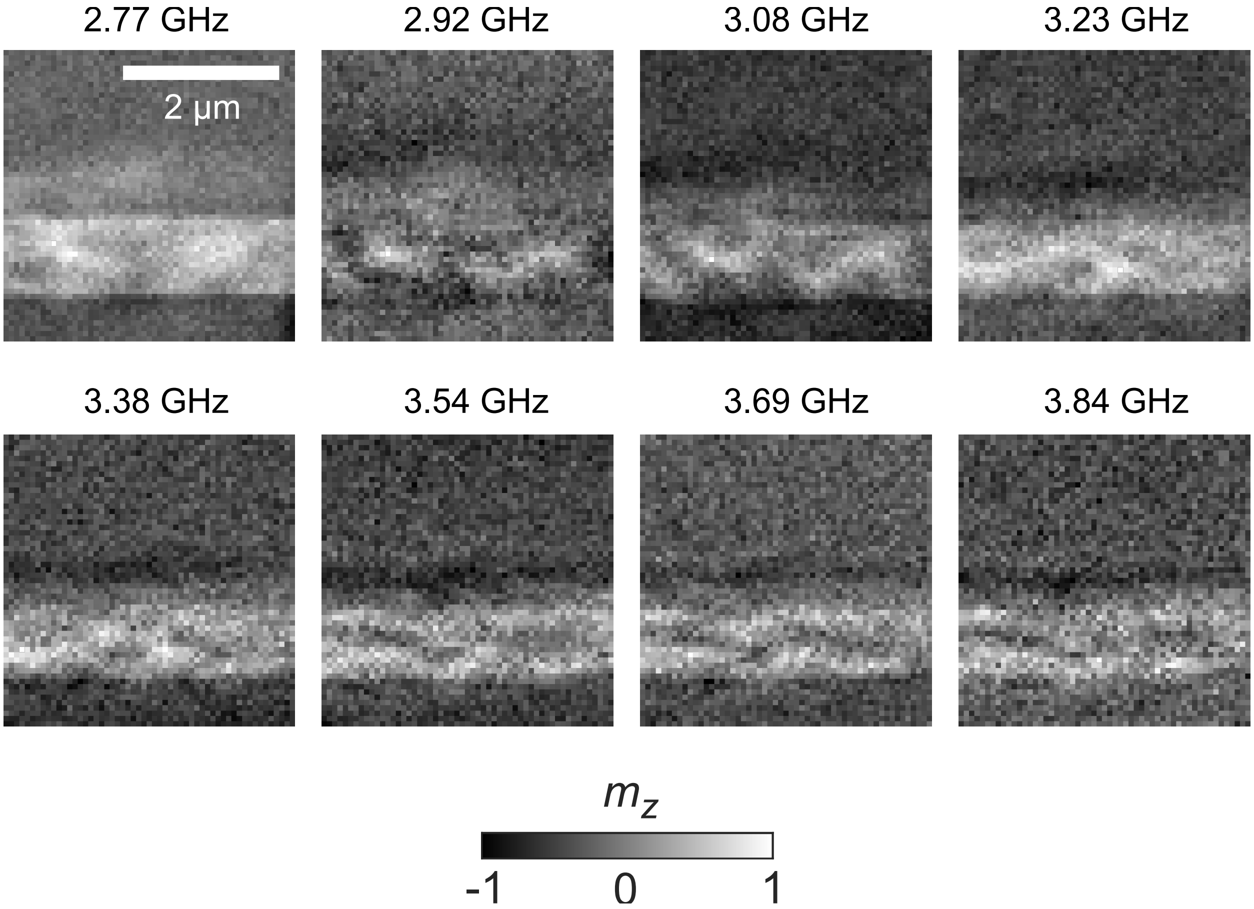

Supplementary Fig. S2: Frequency filtered results of TR-STXM measurements at indicated excitation frequencies, recorded at $T = 190\,\mathrm{K}$, $B = 22.5\,\mathrm{mT}$. Snapshots of the normalized out-of-plane magnetization component m_z, given in arbitrary units. Scalebar applies to all measurements.

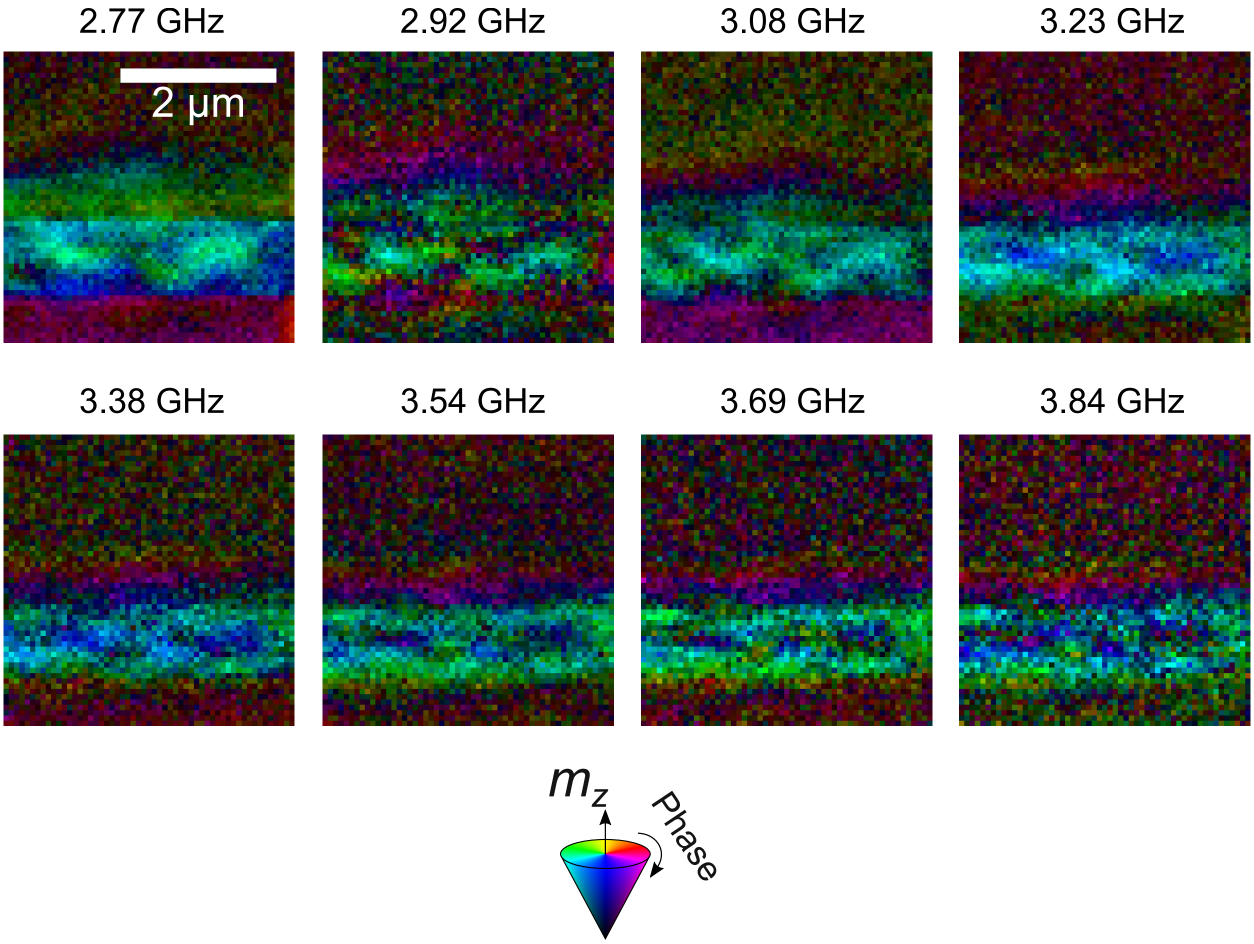

Supplementary Fig. S3: Frequency filtered results of TR-STXM measurements at indicated excitation frequencies in HSV representation, recorded at $T = 190\,\mathrm{K}$, $B = 22.5\,\mathrm{mT}$. Phase and normalized out-of-plane magnetization component m_z are encoded according to the colored cone at the bottom. Scalebar applies to all measurements.

Spin-wave dispersion calculations

The spin-wave dynamics are determined through the utilization of the Landau-Lifshitz (LL) equation of motion, represented as follows:

$$\frac{d\mathbf{M}^{(\nu)}}{dt} = -\mu_0\gamma\mathbf{M}^{(\nu)} \times \mathbf{H}^{\mathrm{e}(\nu)}. \tag{1}$$

In this equation, γ represents the magnitude of the gyromagnetic ratio, $\mathbf{M}^{(\nu)}$ is the magnetization of sublayer ν, and $\mathbf{H}^{\mathrm{e}(\nu)}$ denotes the effective field acting on the ν-th sublayer. A reference coordinate system (x, y, z) is adopted, where the z axis aligns with the equilibrium magnetization, the y axis is perpendicular to the magnetic film, and the x axis lies within the film's plane. It is assumed that the equilibrium magnetization remains uniform across the thickness of the film. For small oscillations of magnetization around the equilibrium state, we can express the magnetization vector and the effective field as follows: $\mathbf{M}^{(\nu)} = M_{s_\nu}\hat{z} + \mathbf{m}$ and $\mathbf{H}^{\mathrm{e}(\nu)} = H^{\mathrm{e}0}_{z_\nu}\hat{z} + \mathbf{h}^{\mathrm{e}}$, respectively. Here, $\mathbf{m} = m_{x_\nu}\hat{x} + m_{y_\nu}\hat{y}$ represents the dynamic magnetization, M_{s_ν} is the saturation magnetization of the ν-th sublayer, and $\mathbf{h}^{\mathrm{e}}$ is directly proportional to $\mathbf{m}$. Therefore, assuming that $\mathbf{m} = \mathbf{m}(x)e^{i\omega t}$ with ω denoting the angular frequency, and disregarding second-order terms in $\mathbf{m}(x)$, the LL equation of motion can be expressed as follows

$$i(\omega/\mu_0\gamma)m_{x_\nu}(x) = -m_{y_\nu}(x)H^{\mathrm{e}0}_{z_\nu} + M_{\mathrm{s}_\nu}h^{\mathrm{e}}_{y_\nu}(x) \tag{2}$$

and

$$i(\omega/\mu_0\gamma)m_{y_\nu}(x) = m_{x_\nu}(x)H^{\mathrm{e}0}_{z_\nu} - M_{\mathrm{s}_\nu}h^{\mathrm{e}}_{x_\nu}(x). \tag{3}$$

Note that the dependence of the dynamic magnetization on the x-coordinate is attributed to the assumption that spin-wave propagation occurs along the x axis (Damon-Eshbach modes). Specifically, this dependence is given by $\mathbf{m}(x) = \mathbf{m}_{\mathbf{k}}e^{ikx}$, where k represents the wave vector. Additionally, equilibrium considerations dictate that $H^{\mathrm{e}0}_{x_\nu} = H^{\mathrm{e}0}_{y_\nu} = 0$. Equations (2) and (3)

can be formulated as an eigenvalue problem, which can be expressed as

$$\tilde{\mathbf{A}}\mathbf{m_k} = i(\omega/\mu_0\gamma)\mathbf{m_k}. \tag{4}$$

The matrix elements of $\tilde{\mathbf{A}}$ are associated with the energetic interactions within the system. In the present scenario, these interactions are Zeeman, demagnetizing, perpendicular anisotropy, in-plane uniaxial anisotropy, and interlayer terms that interconnect the magnetic sublayers. The interlayer energies stem from the bilinear interlayer exchange (with a strength denoted as J) and the dipolar interaction induced by both surface and volumetric dynamic magnetic charges within the opposing sublayers. Detailed information about these interactions and their corresponding matrix elements can be found in Appendix A.3 of Reference 1.

Regarding the interlayer exchange term, the energy density associated with the coupling between sublayers ν and η can be expressed as:

$$\epsilon^{\text{inter}} = -\frac{J}{M_{\mathrm{s}_\nu} M_{\mathrm{s}_\eta}} \mathbf{M}_\nu(\mathbf{r}) \cdot \mathbf{M}_\eta(\mathbf{r}), \tag{5}$$

In situations where the dynamic matrix method employs numerous sublayer divisions, it can be shown that J can be represented as $2A/d$, with d denoting the sublayer thickness.[1,2] Here, A represents the exchange constant associated with the intralayer exchange interaction within the system. Consequently, in cases of isotropic exchange, this exchange constant holds the same value for laterally and vertically coupled magnetic moments. In scenarios involving anisotropic exchange, the exchange interaction between laterally coupled spins is proportional to A, whereas along the normal direction, the spins are coupled by a constant A' (or $J' = 2A'/d$). As such, the dynamic matrix method facilitates the consideration of the anisotropic nature of the exchange interaction, as detailed in the main text of the manuscript.

Additional Model Calculations

In this section, additionally calculated results for different parameter sets are presented. The standard set of parameters used for all simulations, if not specified otherwise, are $B_{\mathrm{ext}} = 22.5\,\mathrm{mT}$, $M_{\mathrm{s}} = 210\,\mathrm{kA\,m^{-1}}$, $A_{\mathrm{ex}} = 9\,\mathrm{pJ\,m^{-1}}$, $H_{\mathrm{a}} = 1.6\,\mathrm{kA\,m^{-1}}$, and $J = 16\,\mathrm{mJ\,m^{-2}}$, with the field applied in-plane. The system consists of 29 layers with a spacing of 0.309 nm and a layer thickness of 0.659 nm, resulting in an overall thickness of 28 nm.

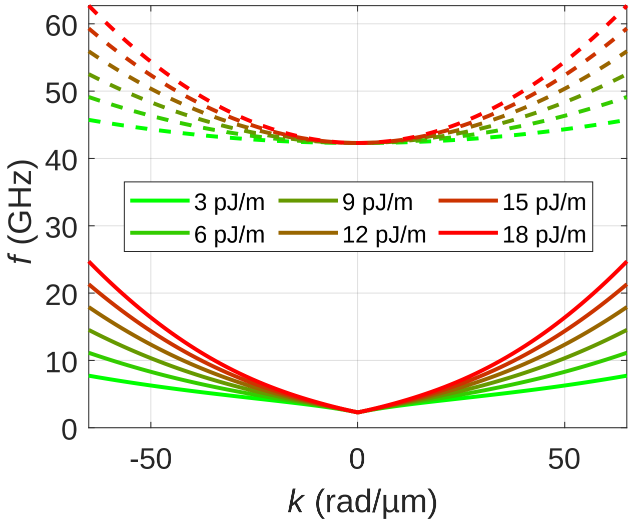

Supplementary Fig. S4: Calculated dispersion relation of 0th order (solid lines) and 1st higher order (dashed lines) Damon-Eshbach type spin-waves for different values of the exchange stiffness A_{ex}. All other parameters are those of standard Fe_5GeTe_2.

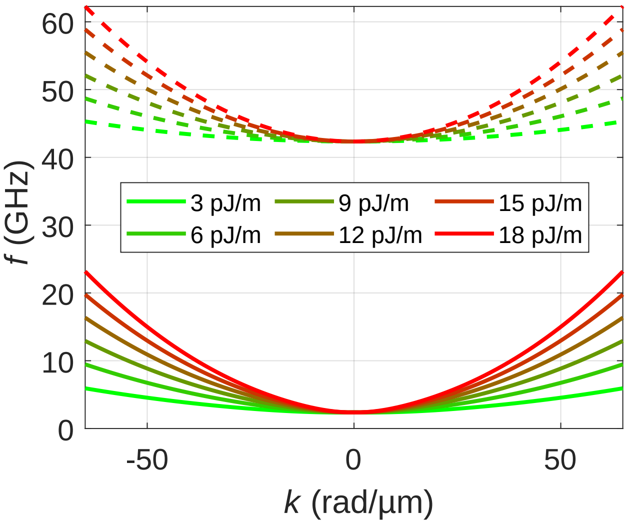

Supplementary Fig. S5: Calculated dispersion relation of 0th order (lines) and 1st higher order (dashed lines) backward volume type spin-waves for different values of the exchange stiffness A_{ex}. All simulation parameters are those of standard Fe_5GeTe_2.

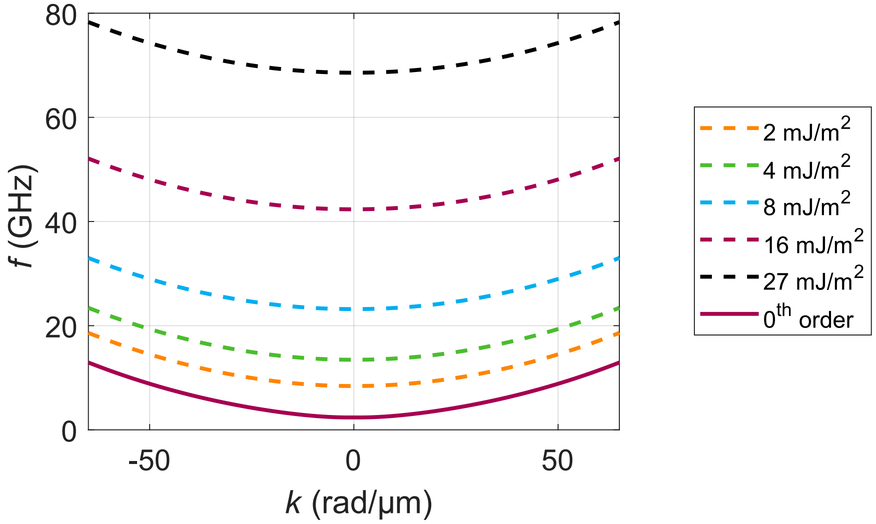

Supplementary Fig. S6: Calculated dispersion relation of the 0th order (purple solid line) and 1st higher order (dashed lines) backward volume type spin-waves for different values of interlayer coupling strength J_{inter}. All other parameters are those of standard Fe_5GeTe_2.

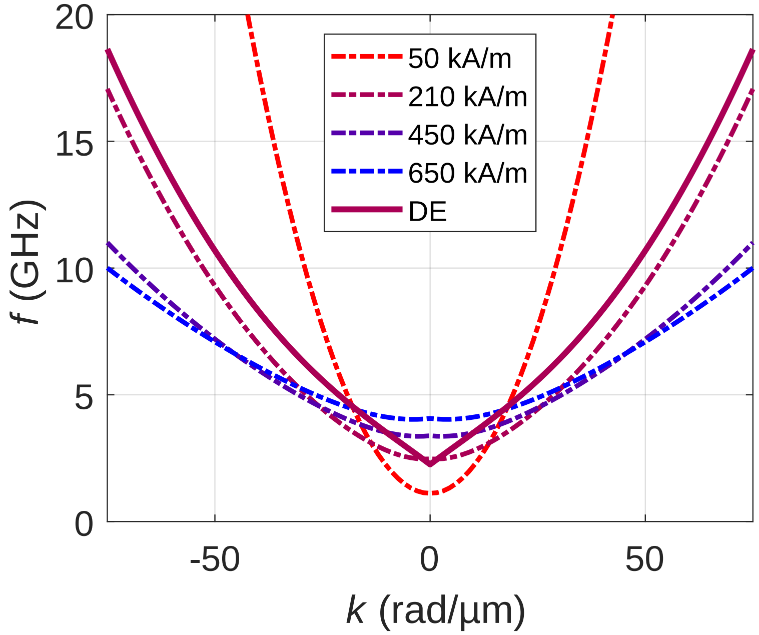

Supplementary Fig. S7: Calculated dispersion relation of backward volume type spin-waves for different values of the saturation magnetization M_s, DE dispersion at $M_s = 210\,\mathrm{kA\,m^{-1}}$ for reference. All other parameters are those of standard Fe_5GeTe_2.

References

(1) R. A. Gallardo, P. Alvarado-Seguel, T. Schneider, C. Gonzalez-Fuentes, A. Roldán-Molina, K. Lenz, J. Lindner, and P. Landeros, New J. Phys. 21, 033026 (2019).

(2) V. Sluka, T. Schneider, R. A. Gallardo, A. Kákay, M. Weigand, T. Warnatz, R. Mattheis, A. Roldán-Molina, P. Landeros, V. Tiberkevich, A. Slavin, G. Schütz, A. Erbe, A. Deac, J. Lindner, J. Raabe, J. Fassbender, and S. Wintz, Nat. Nanotech. 14, 328 (2019).
